# Changes in Gingival Crevicular Fluid Endocan (ESM-1) Levels as a Potential Biomarker After Non-Surgical Periodontal Treatment in Periodontitis Patients

**DOI:** 10.3390/biomedicines13051159

**Published:** 2025-05-09

**Authors:** Bilge Karci, Kevser Sokmen

**Affiliations:** Department of Periodontology, Faculty of Dentistry, Alanya Alaaddin Keykubat University, 07400 Alanya, Türkiye; kevser.sokmen@alanya.edu.tr

**Keywords:** endocan, ESM-1, periodontitis, vascular endothelial growth factor A, tumor necrosis factor-alpha, gingival crevicular fluid

## Abstract

**Background**: This study aimed to investigate endocan (ESM-1) levels in periodontitis patients before and after non-surgical periodontal treatment by analyzing the relationship between vascular endothelial growth factor A (VEGF-A) and tumor necrosis factor-alpha (TNF-α) in gingival crevicular fluid (GCF). **Methods**: This study included 26 periodontally healthy people as controls (Group 1) and 27 patients with Stage III-Grade B periodontitis (Group 2). Demographic and periodontal variables were assessed. GCF samples were collected from every subject both before and 6 weeks following non-surgical periodontal therapy (NSPT). Using an enzyme-linked immunosorbent test, biomarker levels were determined. **Results**: The periodontitis patients showed higher ESM-1 levels than the controls, although the difference was not significant (*p* > 0.005). The ESM-1 levels decreased significantly after treatment (*p* = 0.001). The VEGF-A levels did not differ significantly between the periodontitis patients and controls (*p* > 0.005) and decreased non-significantly following treatment (*p* > 0.005). The TNF-α levels were significantly higher in the periodontitis patients than the controls (*p* = 0.000) and decreased significantly after treatment (*p* = 0.000). A significant correlation was found between TNF-α and both probing depth (PD) and interproximal clinical attachment level (iCAL) in the control group (*p* < 0.05). In the periodontitis group, the VEGF levels were significantly correlated with the gingival index (GI) (*p* < 0.05). Significant correlations were identified between ESM-1 and VEGF-A and ESM-1 and TNF-α, as well as VEGF-A and TNF-α, in both the control group and following treatment (*p* < 0.05). **Conclusions**: ESM-1 and TNF-α levels decreased with non-surgical periodontal treatment in GCF. Within the limits of the study, the findings suggest that ESM-1 levels in periodontal tissues may be an indicator of periodontal disease.

## 1. Introduction

Periodontitis is a chronic inflammatory disease that destroys the tissue that supports teeth [1]. Vascular dilatation, papillary permeability, and leukocyte extravasation are part of the inflammatory process, which occurs as the body reacts to pathogens. Polymorphonuclear leukocytes (PMNLs) are the first cells to arrive at the site of inflammation, while leukocyte migration against microbial biofilm is a process that begins with endothelial cells [2]. In the gingival sulcus, PMNLs participate in the immunological response to periodontopathogens during the initial stage of host defense [3]. The inflammatory response increases with cytokine and chemokine production [4]. Periodontal soft tissues, such as the gingiva, play protective and supportive roles in infection and trauma cases. The fibroblasts of periodontal structures produce and organize collagens, fibronectin, and other proteoglycans, greatly contributing to tissue repair mechanisms [5].

Instead of relying exclusively on clinical indicators, gingival crevicular fluid (GCF) may be employed to characterize a site at the molecular level because it is a site-specific exudate that reflects the inflammatory process pathologically [6].

Tumor necrosis factor-alpha (TNF-α) is a key proinflammatory cytokine that plays a critical role in the inflammatory response within periodontal tissues [7]. Ligands for the receptor activator of nuclear factor-kappa B and matrix metalloproteinases are secreted by TNF-α, which is linked to bone resorption and connective tissue deterioration [8]. Regarding periodontal disease, TNF-α is a crucial biomarker that may facilitate diagnosis, prognosis, and treatment. GCF TNF-α levels rise in inflammatory diseases like periodontitis, aiding in the degradation of inflamed periodontal tissues [9].

Angiogenesis plays a crucial role in the progression of inflammatory disorders [10]. Although many growth factors and cytokines contribute to controlling angiogenesis, the most effective element that targets the vascular endothelium is the vascular endothelial growth factor (VEGF) [11]. The active degradation of periodontal structures has been linked to increased TNF-α in the GCF, and this mediator is a major stimulator of VEGF. Thus, by enhancing vascular permeability and angiogenesis, VEGF can accelerate the course of periodontal disease [12].

The role of VEGF in gingival inflammation is multifaceted. It not only promotes angiogenesis but also contributes to the recruitment of inflammatory cells to the site of inflammation. The increased vascularity facilitated by VEGF allows the infiltration of leukocytes, which release proinflammatory cytokines and mediators, further exacerbating the inflammatory response [13,14].

Endothelial cells secrete a variety of chemicals, one of which is endothelial cell-specific molecule-1 (ESM-1), also known as endocan [15]. ESM-1 is a soluble dermatan sulfate proteoglycan secreted by activated endothelium, with its secretion being influenced by proinflammatory cytokines [16]. ESM-1 participates in numerous significant biological processes, including cell transformation, cell proliferation, migration, vascularization, and tumor metastasis [15,17,18].

ESM-1 overexpression has been observed in inflammatory disorders, cardiovascular illnesses, sepsis, cancer, and obesity [15,18,19]. Inflammation is known to play a significant role in the pathogenetic alterations of periodontitis, and endocan plays a crucial regulatory role in angiogenesis and inflammatory reactions [20]. The expression of VEGF may determine the angiogenic effects of endocan. According to some research, VEGF can increase endocan activity [17,21,22]. Endocan expression is also greatly increased by inflammatory signaling pathways when proinflammatory cytokines such as TNF-α are activated [23]. This leads to inflammatory reactions mediated by TNF-α released by monocytes or macrophages [20,24,25].

Endocan plays a pivotal role in the pathogenesis of periodontal disease by modulating both inflammatory and angiogenesis-related pathways. Its ability to upregulate proinflammatory cytokines through the TLR2-MAPK-NFkB pathway and its correlation with adhesion molecules such as intercellular adhesion molecule-1 (ICAM-1) and lymphocyte-function-associated antigen-1 (LFA-1) highlight its role in inflammation [26,27].

The rationale behind this study stems from the need to identify reliable, site-specific biomarkers that can reflect the molecular dynamics of periodontal inflammation and its resolution following therapy. While traditional clinical parameters provide valuable information about disease status, they do not capture the underlying biological processes at the tissue level. Although elevated ESM-1 levels have been observed in various systemic inflammatory diseases, its role in periodontal disease, particularly in relation to established markers like TNF-α and VEGF-A, remains insufficiently explored.

This study aimed to compare endocan levels in individuals with periodontitis both before and after non-surgical periodontal therapy (NSPT) by analyzing the interaction between the proinflammatory mediator TNF-α and the angiogenic factor VEGF-A.

## 2. Materials and Methods

### 2.1. Study Population

The current investigation was conducted from February 2020 to September 2020 at the Alanya University Dentistry Faculty, Periodontology Department, Antalya, Türkiye. Participants who requested dental treatment or gingival evaluation at the periodontology outpatient clinic were assigned to the examination. This study included 26 periodontally and systemically healthy people as controls (Group 1) and 27 people with Stage III-Grade B periodontitis who were systemically healthy (Group 2). The investigation complied with the Declaration of Helsinki, as updated in 2013, and was approved by the ALKU Faculty of Medicine Ethics Committee (date: January 2020, Protocol No. 15-02). The investigation’s objective and methodology were elucidated to each participant. Written informed consent was acquired from each participant.

### 2.2. Inclusion and Exclusion Criteria

Smokers, pregnant and breastfeeding women, and individuals with systemic disorders (including obesity, rheumatoid arthritis, and diabetes mellitus) were excluded from the study. Additionally, individuals who had undergone any periodontal therapy or used antibiotics or anti-inflammatory medication in the previous 6 months were excluded. Thorough, full-mouth clinical, periodontal, and radiographic assessments were used to determine the selection requirements. All participants were in a state of systemic health. Participants devoid of allergies and inflammatory or autoimmune conditions were also incorporated into the study.

### 2.3. Periodontal Examination

A full-mouth clinical, radiographic, periodontal test was used to evaluate the following inclusion criteria: gingival index (GI) [28], plaque index (PI) [29], interproximal clinical attachment level (iCAL), bleeding on probing (BOP) [30], and probing depth (PD). Individuals were required to possess at least 20 natural teeth, excluding the third molars. According to the most recent classification by AAP and EEP, all individuals were diagnosed with Stage III and Grade B periodontitis with a percentage of bone loss per age between 0.25 and 1.0, with 30% or more sites showing a PD > 5 mm and iCAL ≥ 5 mm [31]. BOP < 10% and PD ≤ 3 mm were indicators of periodontal health (control group). Only millimeter-level values that were more than 90% identical between the baseline and 48 h points were included [32]. To assess the intra-rater reliability of measurements taken by the same examiner 48 h apart, the intraclass correlation coefficient (ICC), which is appropriate for continuous data, was calculated. The resulting ICC value was 0.921, indicating excellent consistency between measurements [33].

### 2.4. GCF Collection

GCF was collected the day after the measurements to prevent its contamination with blood. For the periodontitis group, specimens were taken from the mesial areas of single-rooted teeth with PPD ≥ 5 mm. Single-rooted teeth were selected randomly in the control group. The sample collection site was left in isolation, and steering clear of the marginal gingiva, supragingival plaque was excised with a curette. Paper strips (Oraflow Inc., Sarasota, FL, USA) were employed to gather GCF samples. The strips were placed inside the groove and held there for 30 s when some stiffness was felt [34]. Strips with a noticeable bloodstain were not included. Phosphate-buffered saline (200 µL) was added to the microcentrifuge tubes holding the paper strips. The tubes were stored at −80 °C until analysis.

### 2.5. Periodontal Therapy

NSPT was started and completed by the same researcher (BK). Following the collection of baseline GCF data, the patients with periodontitis were given NSPT, which included teaching them proper dental hygiene practices and performing scaling and root planing (SRP) using curettes and manual scalers. Each patient received SRP once a week, arranged in quadrants. Antibiotics were not part of the therapy plan. Local anesthetic was administered when needed. The control group only received advice on brushing and flossing. The dental hygiene instructions that were reviewed weekly throughout the study included the modified Bass technique, which comprised both the brushing approach and dental flossing. The patients with periodontitis were re-evaluated for 6 weeks following the end of periodontal therapy, and further GCF samples were collected. GCF samples were retrieved from their first location at the mesial site of single-rooted teeth with PD ≥ 5 mm to standardize site-specific inflammatory responses. The clinical efficacy of NSPT was assessed using PD reduction, CAL, and BOP% as primary endpoints, following the evidence-based thresholds proposed by Cobb et al. [35].

### 2.6. GCF ELISA Method for Endocan, TNF-α, and VEGF

As mentioned before, VEGF-A, TNF-α, and ESM-1 values in the GCF were measured with a sandwich enzyme-linked immunosorbent assay (Bioassay Technology Laboratory, Shanghai, China) following the manufacturer-provided methodology [36]. The total quantity of GCF samples was ascertained from standard curves.

The VEGF-A test had an analytical sensitivity of 10.42 ng/L and a calibration range of up to 6000 ng/L. The intra-assay coefficient of variation (CV) was <8%, and the inter-assay CV was <10%. The TNF-α test had an analytical sensitivity of 1.52 ng/L and a calibration range of up to 900 ng/L. Both the intra- and inter-assay CVs were <10%. The ESM-1 test had an analytical sensitivity of 2.56 ng/L and a calibration range of up to 2000 ng/L. The intra-assay CV was <8%, while the inter-assay CV was <10%.

### 2.7. Statistical Analysis

The sample size was calculated as 52 for an effect size of 0.85, a power of 0.95, and a significance level of 0.05. The Shapiro–Wilk and Kolmogorov–Smirnov tests were employed to assess whether the data were normally distributed. Clinical and biochemical values were compared between groups by nonparametric tests. If the data were not normally distributed, Mann–Whitney U tests were applied for post hoc group analysis. The Wilcoxon test was used to evaluate paired biochemical and periodontal data (baseline and 6 weeks). The relationship between GCF endocan, TNF-α, and VEGF-A values and clinical periodontal markers was examined using the Spearman rank correlation test. Statistical analysis was performed utilizing SPSS Ver. 22.0, and *p* scores < 0.05 indicated statistical significance.

## 3. Results

### 3.1. Clinical Parameters

Table 1 and Table 2 summarize the participants’ clinical periodontal assessments and demographic characteristics. Age and sex did not significantly differ between the groups (*p* > 0.05). Group 2 exhibited a statistically significant increase in all clinical measures compared to Group 1 (*p* < 0.05). Six weeks following NSPT, the patients in Group 2 showed a substantial decrease in all clinical measures in the complete mouth (*p* < 0.05).

### 3.2. Biochemical Findings

The total amounts of VEGF-A, TNF-α, and ESM-1 are displayed in Table 3. The total ESM-1 values were elevated in Group 2 compared with Group 1, but this difference was not significant (*p* > 0.05). Following therapy, a significant decrease in ESM-1 values occurred (*p* = 0.001; Table 4). There was no significant difference in total VEGF-A levels in the GCF between the two groups (*p* > 0.05). The levels of VEGF-A decreased in the periodontitis patients following therapy. However, no statistically significant difference was noted (*p* > 0.05; Table 4 and Table 5). The total TNF-α score was considerably greater in Group 2 patients than in Group 1 (*p* = 0.000). After receiving therapy for periodontitis, TNF-α levels statistically significantly decreased (*p* = 0.000; Table 3 and Table 4).

### 3.3. Correlations

Table 6 shows the correlation coefficients of the GCF contents of ESM-1, VEGF-A, and TNF-α with the periodontal clinical parameters. The total level of TNF-α in the control group was statistically significantly correlated with both PPD and iCAL (*p* < 0.05). The VEGF values and GI were shown to be significantly correlated in the periodontitis group (*p* < 0.05). The ESM-1 values were shown to be significantly correlated with VEGF-A (*p* < 0.05), as well as TNF-α (*p* < 0.05), and the VEGF-A values correlated with the TNF-α values (*p* < 0.05), in both the control and post-therapy periodontitis groups.

## 4. Discussion

This study assessed the levels of ESM-1, VEGF-A, and TNF-α in GCF in the context of periodontal disease prior to and following NSPT. The hypothesis was that ESM-1, VEGF-A, and TNF-α values would be higher in the periodontitis group than in the control group and would decrease after NSPT. However, the VEGF-A and ESM-1 levels in both groups and the VEGF-A levels before and after periodontal therapy were not found to be statistically significant. This may be related to the severity and grade of periodontitis.

Serum-like GCF, which is found in the gingival sulcus, contains inflammatory cells and mediators in people with periodontitis as opposed to healthy tissue, and its content is linearly connected with the degree of inflammation. Consequently, it serves as an advantageous diagnostic instrument to identify the pathological alterations associated with periodontal disease [6].

GCF and its inflammation-related compounds have diagnostic and prognostic utility in evaluating the pathophysiology of periodontal disorders [37]. Preceding research showed that biomarkers were more accurately measured when expressed as total GCF quantities in the GCF for each sample period, as opposed to concentrations [38,39]. Consequently, in our investigation, biomarkers were quantified as total amounts.

VEGF-A can enhance vascular permeability, perhaps exacerbating inflammation during the initial phases of periodontal problems. The involvement of VEGF in the pathophysiology of periodontal problems seems inconsistent, with VEGF expression reported as increasing [40,41,42,43], reducing [44], or remaining unchanged [11] during the disease process. In our study, no significant difference in total VEGF-A levels between the periodontitis and control groups was noted. Pradeep et al. [40] and Jayara et al. [41] observed that VEGF-A levels in the GCF of periodontitis patients were statistically significantly reduced following NSPT. However, Afacan et al. [45] determined no statistically significant difference in total VEGF-A levels in the GCF with advanced periodontitis following NSPT. Our study found no statistically significant difference in total VEGF-A levels after NSPT in the periodontitis group. It is likely that these differences in the study results are because VEGF expression may be linked to both the preservation of periodontal health and periodontal tissue destruction [46,47]. Furthermore, our study focused on Stage III-Grade B periodontitis, whereas other studies investigated different classifications. This may explain the difference in the VEGF-A results between them.

In contrast to the investigations of VEGF-A, the results concerning TNF-α in the pathophysiology of periodontal diseases were not contradictory. Periodontal disease is clearly related to elevated TNF-α levels [48]. Like other studies, the current results demonstrated that the periodontitis group had a significantly higher TNF-α level in the GCF. Moreover, the expected fall in TNF-α values in the GCF after periodontal therapy was noted.

Türer et al. and Tayman et al. found GCF ESM-1 levels to be significantly higher in the periodontitis group than in the control group [27,49]. In our study, ESM-1 levels were higher in the periodontitis group than in the control group, but this difference was not statistically significant. This may be due to the stage of periodontitis.

Türer et al. and Kumar et al. found that GCF ESM-1 and TNF-α levels decreased significantly after NSPT in the periodontitis group [49,50]. Similarly, in this study, we found that after NSPT, the levels of TNF-α and ESM-1 decreased. This could be because endothelium function improves and the proinflammatory influence of ESM-1 gradually decreases. The reduction in GCF ESM-1 and TNF-α levels after NSPT may have possible prognostic relevance in assessing ESM-1 levels throughout periodontal therapy.

Furthermore, the clinical periodontal markers exhibited a considerable reduction following NSPT in comparison to pretreatment levels. This indicates that periodontal treatment is effective; furthermore, inflammation was diminished.

The correlations between GCF VEGF-A, ESM-1, and TNF-α levels and periodontal disease were also assessed. TNF-α significantly correlated with PPD and iCAL in the control group. VEGF levels and GI were significantly correlated in the periodontitis group. ESM-1 levels were significantly related to TNF-α and VEGF-A, while VEGF-A and TNF-α values were also correlated in both the control and post-treatment periodontitis groups. Based on these results, ESM-1 may have an inflammatory effect in periodontitis.

This study’s limitations include its small sample size, short follow-up period, and focus on Stage III-Grade B periodontitis patients.

Ultimately, these findings suggest that reduced levels of TNF-α and ESM-1 in periodontitis patients following NSPT may be associated with ESM-1 and periodontal inflammation.

## 5. Conclusions

This study’s results indicated that GCF ESM-1 levels were elevated but not statistically significant in periodontitis patients; these levels, along with TNF-α, were considerably reduced following NSPT. Limited literature is available about the relationship between ESM-1 and periodontitis. Future studies should include larger sample sizes and more diverse patient populations, as well as various stages and grades of periodontitis and different systemic conditions, to enhance the generalizability of findings and clarify the relationship between ESM-1 and periodontal disease severity. Long-term, multicenter studies are needed to evaluate the dynamics of ESM-1 levels over extended periods and in response to different periodontal therapies.

## Figures and Tables

**Table 1:** Demographic characteristics of the study groups.

		Group 1 (Control) (n = 26)	Group 2 (Periodontitis) (n = 27)
Age (mean ± SD)		41.5 ± 7.5	45.5 ± 9.0
Sex	Male	13 (50%)	15 (55.6%)
	Female	13 (50%)	12 (44.4%)

**Table 2:** Clinical periodontal parameters of the study groups.

	Group 1 (C)	Group 2 (Before Treatment; BT)	Group 2 (After Treatment; AT)	*p*-Value
PI	0.73 ± 0.45	2.55 ± 0.64	0.85 ± 0.60	<0.05 (BT-AT, BT-C)
GI	0.69 ± 0.47	2.44 ± 0.57	0.88 ± 0.64	<0.05 (BT-AT, BT-C)
PD (mm)	2.26 ± 0.66	5.77 ± 1.01	3.70 ± 1.10	<0.05 (BT-AT, BT-C, AT-C)
BOP (%)	0.00 ± 0.00	73.33 ± 8.77	10.55 ± 4.87	<0.05 (BT-AT, BT-C, AT-C)
iCAL (mm)	2.26 ± 0.66	6.29 ± 1.48	4.22 ± 1.50	<0.05 (BT-AT, BT-C, AT-C)

Values are presented as mean ± SD. Mann–Whitney U test (unpaired observations). Wilcoxon signed-rank test (paired observations). *p* < 0.05 indicates a statistically significant difference. Abbreviations in parentheses represent groups with statistically significant differences.

**Table 3:** GCF endocan, VEGF-A, and TNF-α levels.

Parameter	Group 1 (Control)	Group 2 (Before Treatment; BT)	Group 2 (After Treatment; AT)
ESM-1 (ng/30 sn)	1686.22 ± 199.20	1706.71 ± 195.65	1541.27 ± 178.62
TNF-α (ng/30 sn)	209.09 ± 17.16	236.51 ± 22.00	188.77 ± 17.36
VEGF-A (ng/30 sn)	1601.06 ± 169.25	1695.76 ± 184.00	1424.70 ± 172.47

Data are presented as the mean ± SD.

**Table 4:** Pairwise comparisons of biochemical levels.

		t	df	*p*-Value
ESM-1	Group 2 (BT)-Group 2 (AT)	3.55	26	0.001 *
	Group 1-Group 2 (AT)	2.791	51	0.007 *
	Group 1-Group 2 (BT)	−0.378	51	0.707
TNF-α	Group 2 (BT)-Group 2 (AT)	43.798	26	0.000 *
	Group 1-Group 2 (AT)	8.787	51	0.000 *
	Group 1-Group 2 (BT)	5.069	51	0.000 *
VEGF-A	Group 1-Group 2 (BT)	1.951	51	0.057

* *p* < 0.05 indicates a statistically significant difference. Independent group *t*-test (unpaired observations). Paired samples *t*-test (paired observations).

**Table 5:** Pairwise comparisons of biochemical levels (continued).

		z	*p*
VEGF-A	Group 1-Group 2 (AT)	−4.715	0.000 *
	Group 2 (BT)-Group 2 (AT)	3.027	0.002

* *p* < 0.05; statistically significant difference. Mann–Whitney U test (unpaired observations). Wilcoxon signed-rank test (paired observations).

**Table 6:** Correlations between GCF ESM-1, VEGF-A, TNF-α levels and periodontal clinical parameters.

	Group 1 (Control)		Group 2 (Before Treatment; BT)		Group 2 (After Treatment; AT)	
Parameter	r	*p*	r	*p*	r	*p*
ESM-1 to GI	0.144	0.481	−0.079	0.696	0.061	0.761
ESM-1 to PPD	0.118	0.565	−0.008	0.968	0.026	0.899
ESM-1 to iCAL	0.118	0.565	0.038	0.851	−0.064	0.753
VEGF-A to GI	0.111	0.589	−0.446 *	0.020 *	0.105	0.601
VEGF-A to PPD	0.208	0.307	−0.023	0.909	0.277	0.161
VEGF-A to iCAL	0.208	0.307	0.075	0.709	0.133	0.509
TNF-α to GI	−0.033	0.872	0.029	0.886	0.159	0.429
TNF-α to PPD	0.414 *	0.035 *	0.067	0.740	0.032	0.874
TNF-α to iCAL	0.414 *	0.035 *	0.074	0.712	0.096	0.635
ESM-1 to VEGF-A	0.491 *	0.011 *	0.247	0.215	0.599 *	0.001 *
ESM-1 to TNF-α	0.529 *	0.005 *	0.262	0.187	0.593 *	0.001 *
VEGF-A to TNF-α	0.399 *	0.043 *	0.298	0.131	0.417 *	0.030 *

Pearson’s correlation, r. * Significant at the 0.05 level.

## Data Availability

The data can be provided upon reasonable request to the corresponding author. The data presented in this study are available on request from the corresponding author. The data are not publicly available due to privacy.

## Abbreviations

The following abbreviations are used in this manuscript:

ESM-1	Endothelial cell-specific molecule-1
GCF	Gingival crevicular fluid
NSPT	Non-surgical periodontal therapy
PMNL	Polymorphonuclear leukocytes
TNF-α	Tumor necrosis factor-alpha
VEGF	Vascular endothelial growth factor
PI	Plaque index
GI	Gingival index
iCAL	Interproximal clinical attachment level
BOP	Bleeding on probing
PD	Probing depth
CV	Coefficient of variation
ICAM-1	Intercellular adhesion molecule-1
LFA-1	Lymphocyte-function-associated antigen-1
